# The Outcome after Laser Therapy of Monochorionic Twin Pregnancies Complicated by Twin-Twin Transfusion Syndrome with Coexistent Selective Fetal Growth Restriction

**DOI:** 10.3390/jcm13082432

**Published:** 2024-04-21

**Authors:** Javier U. Ortiz, Johanna Guggenberger, Oliver Graupner, Eva Ostermayer, Bettina Kuschel, Silvia M. Lobmaier

**Affiliations:** Division of Obstetrics and Perinatal Medicine, Department of Obstetrics and Gynecology, University Hospital Rechts der Isar, Technical University of Munich, Ismaninger Strasse 22, 81675 Munich, Germany; johanna.guggenberger@mri.tum.de (J.G.); oliver.graupner@mri.tum.de (O.G.); eva.ostermayer@mri.tum.de (E.O.); bettina.kuschel@mri.tum.de (B.K.); silvia.lobmaier@mri.tum.de (S.M.L.)

**Keywords:** twin-twin transfusion syndrome, selective fetal growth restriction, laser therapy

## Abstract

**Background:** Most previous studies evaluated outcomes of twin–twin transfusion syndrome (TTTS) without considering the coexistence of selective fetal growth restriction (sFGR). The objectives of this study were to compare twin survival and pregnancy complications after laser therapy of TTTS with and without sFGR. **Methods:** For this purpose, a retrospective cohort study including 98 monochorionic diamniotic twins and three dichorionic triamniotic triplets treated in a single tertiary center was conducted. **Results:** Overall, 46 twins had selective fetal growth restriction (26 type I, 13 type II, 7 type III). At birth, donor survival (61% vs. 91%), double survival (57% vs. 82%), and overall survival (75% vs. 88%) were significantly lower in the group with coexistent sFGR. Recipient survival (89% vs. 86%), miscarriage (7% vs. 2%), PPROM < 32 weeks (48% vs. 29%), and preterm delivery < 32 weeks (52% vs. 45%) were not significantly higher in the group with coexistent sFGR. Donor twins with sFGR type I (69% vs. 91%) and types II–III (50% vs. 91%) showed significantly lower survival than those without sFGR. Multivariate regression analysis identified sFGR and its subtypes as independent predictors of donor demise. **Conclusions:** the coexistence of sFGR in TTTS pregnancies was associated with poor donor outcomes and is probably the most important predictor of donor survival.

## 1. Introduction

Monochorionic (MC) twin pregnancies represent a single condition in which two fetuses share a placenta and are connected by vascular anastomosis [1]. Chronic unbalanced blood transfusion through arteriovenous anastomosis leads to hypovolemia, oliguria, and oligohydramnios in one twin (donor) and hypervolemia, polyuria, and polyhydramnios in the other twin (recipient). This is called twin–twin transfusion syndrome (TTTS) and occurs in approximately 10–15% of MC twin pregnancies. In addition, unequal sharing of the placenta results in decreased growth and, thus, the small size of one of the twins [2]. This selective fetal growth restriction (sFGR) occurs in about 10% of MC twins. Its natural history is influenced by the discordance of placental territories as well as by the size and number of placental anastomoses. Therefore, TTTS and isolated sFGR represent considerable hemodynamic changes in both twins and are associated with poor outcomes. If one twin dies, its co-twin will also die in 15% of the cases [3]. Furthermore, 25% of those who survive will have a neurological handicap.

While laser photocoagulation of placental vascular anastomosis (LPCV) treats the cause and improves pregnancy outcome in TTTS, there is no causal therapy and no solid evidence on the management of isolated sFGR. It ranges from expectant management to LPCV, or selective cord occlusion of the smaller twin.

The vast majority of previous series described the outcome of TTTS after laser therapy without taking into account the possible coexistence of sFGR, which would negatively affect pregnancy outcome [4,5,6,7].

Our main objective was to compare the survival of MC twins complicated by TTTS with and without sFGR after laser treatment. As a secondary objective, we aimed to compare pregnancy complications between the groups.

## 2. Materials and Methods

### 2.1. Study Protocol

This study was a retrospective analysis of prospectively collected data, including a cohort of consecutive MC diamniotic (DA) twin pregnancies and dichorionic triamniotic (DCTA) triplet pregnancies complicated by TTTS stages I (with maternal orthopnea, uterine contractions or cervical shortening) to IV between 15 and 27 weeks of gestation treated with laser by two fetal surgeons (J.U.O., S.M.L.) at the University Hospital “Klinikum rechts der Isar” of the Technical University of Munich between January 2015 and October 2023. Of all triplets, only MCDA twin pairs were considered. A twin pregnancy was considered MCDA when a T sign with a single placental mass was observed during the first trimester scan [8]. Pregnancies were dated using the larger crown-rump length at the first trimester [9]. A detailed ultrasound scan was performed in both twins to assess fetal condition within 24 h before fetal surgery using a Voluson E8 or E10 (GE Medical Systems, Solingen, Germany) with a 4- to 6-MHZ curvilinear abdominal transducer. TTTS was defined as the deepest vertical pocket of amniotic fluid < 2 cm in the donor’s sac and ≥8 cm before 20 weeks of gestation or ≥10 cm after 20 weeks of gestation in the recipient’s sac [10]. The severity of TTTS was classified according to the Quintero staging system [11]. Umbilical artery (UA) pulsatility index (PI), middle cerebral artery (MCA) PI, and ductus venosus (DV) PI were performed in both twins as previously described [12,13,14]. sFGR was defined as an estimated fetal weight (EFW) < 3rd percentile in one twin or the presence of at least two out of the following four contributory parameters: EFW < 10th percentile in one twin, abdominal circumference < 10th in one twin, intertwin EFW discordance ≥ 25%, UA-PI > 95th percentile in the smaller twin [15]. The EFW included measurements of biparietal diameter, head circumference, abdominal circumference, and femur length. It was calculated using the Hadlock formula [16]. The EFW percentile was determined using a fetal growth chart for MCDA twins [17]. Intertwin EFW discordance was calculated as the difference between the EFW of the larger twin and the smaller twin divided by the EFW of the larger twin multiplied by 100. sFGR was classified according to the UA Doppler pattern of the smaller twin that presents positive end-diastolic flow (EDF) in type I and persistently or intermittently absent-reversed EDF (AREDF) in types II and III, respectively [18]. The UA Doppler of the smaller twin was measured at or near placental cord insertion since, according to the author’s experience, in some cases of sFGR type III, the farther away from the placenta the UA is assessed, the more likely it is that intermittent AREDF will not be detected. Exclusion criteria were maternal age < 18 years and cord occlusion of one twin. The study protocol was approved by the local Ethics Committee (2023-411-S-KK).

The LPCV was always carried out at the vascular equator, regardless of its location with respect to the intertwin membrane, as previously described using diode laser and 8–10 F diameter trocars housing 1–2 mm endoscopes and operative channels [10]. Selective LPCV was performed between January 2015 and December 2018 and the Solomon technique (further laser coagulation of the placenta between the coagulated communicating vessels) since January 2019 [19]. After laser therapy, amniotic fluid was drained until the deepest volume pocket was less than 8 cm. All procedures were carried out under antibiotic prophylaxis (cefuroxime 1.5 g or clindamycin 600 mg intravenous), intravenous sedation (remifentanil 0.05–0.15 µg/kg/min), and local anesthesia (mepivacaine hydrochloride 50 mg). Fetal lung maturation (two 12-mg doses of betamethasone intramuscularly 24 h apart) was administered if gestational age (GA) at surgery was ≥24 weeks. Perioperative tocolysis (atosiban as per protocol intravenous or nifedipine 20 mg oral) was given if at least one of the following criteria was present: regular uterine contractions, cervical length < 25 mm, GA at surgery ≥ 24 weeks. After fetal surgery, ultrasound follow-up was performed within 48 h. The patient was discharged if asymptomatic. Ultrasound controls were carried out weekly for two weeks and at least every two weeks thereafter by referring physicians in most cases.

### 2.2. Analyzed Data

The following data were collected: maternal age, ethnicity, body mass index (BMI), parity, placental location, GA at diagnosis, TTTS stage, sFGR type, UA Doppler pattern (AREDF), DV Doppler pattern (absent or reversed A-wave (ARF)), EFW, EFW percentile, intertwin EFW discordance, cervical length, GA at surgery, type of surgery, duration of surgery (from insertion to removal of the trocar), miscarriage (spontaneous delivery < 24 weeks of gestation without twin survivors), preterm premature rupture of the membranes (PPROM), GA at delivery, type of delivery, survival at birth, birthweight (BW) of live births, BW percentile [20], intertwin BW discordance of living twin siblings (difference between BW of the larger twin and the smaller twin divided by BW of the larger twin multiplied by 100), and neonatal survival (28 days after birth).

### 2.3. Statistical Analysis

Statistical analysis was performed using the Statistical Package for the Social Sciences software (SPSS 24.0, IBM Corp., Armonk, NY, USA). Data are presented as median (interquartile range p25–p75) or absolute and relative frequencies. Mann–Whitney U tests were used to compare the distributions of continuous variables among the groups. Pearson’s chi-squared test or Fisher’s exact test were carried out to compare categorical data. All tests were two-tailed. *p* values < 0.05 were considered statistically significant. Moreover, univariate logistic regression analyses were performed using maternal (age, ethnicity, BMI, parity), ultrasound (placental location, UA AREDF, DV ARF, TTTS stage III–IV, sFGR, cervical length < 25 mm), and surgical (GA at surgery, Solomon technique, duration of surgery) parameters as independent variables with survival at birth as a binary outcome. Statistically significant variables in the univariate logistic regression were included in the multivariate logistic regression.

## 3. Results

During the study period, 107 pregnancies (104 MCDA twins, three DCTA triplets) complicated by TTTS were treated with fetal surgery at our institution. Of these, six MCDA twin pregnancies were excluded (one case with a maternal age < 18 years, three cases of donor’s cord occlusion because of severe bilateral ventriculomegaly, and two cases of recipient’s cord occlusion because of very close umbilical cord insertions). Therefore, 101 patients treated with lasers were included in the final analysis.

In the entire study population, 54 (53%) patients showed TTTS stages III-IV and 46 (46%) had sFGR (26 (26%) type I, 13 (13%) type II, 7 (7%) type III). Median of intertwin EFW discordance was 19% (IQR 11–28), and GA at surgery was 20.6 (18.3–22.9) weeks (Table 1). The median GA at delivery was 32.0 (28.7–34.9) weeks and overall twin survival, survival of both twins, and survival of at least one twin at birth were 82%, 70%, and 94%, respectively (Table 2). Neonatal survival rates were 78%, 63%, and 92% for all twins, both twins, and at least one twin, respectively (Table 2).

TTTS with sFGR showed significantly higher proportions of TTTS stages III–IV and donor twin with UA AREDF, a lower median EFW percentile of the donor twin, and higher intertwin EFW discordance (Table 1). Proportions of ethnicity, nulliparity, anterior placenta, donor and recipient twins with DV ARF, recipient twins with UA AREDF, cervical length < 25 mm, Solomon technique as well as medians of maternal age, BMI, and EFW percentile of the recipient twin were similar in both groups (Table 1).

Regarding pregnancy outcomes, TTTS with sFGR showed significantly lower proportions of donor twin survival, survival of both twins, and overall twin survival both at birth and in the neonatal period (Table 2). There were no significant differences in miscarriage, PPROM < 32 weeks of gestation, preterm delivery < 32 weeks of gestation, GA at delivery, cesarean section, survival of the recipient twin, or survival of at least one twin between the groups (Table 2).

Subgroup analysis showed that donor twins of TTTS with sFGR type I (69% vs. 91%; *p* = 0.022) and sFGR types II–III (50% vs. 91%; *p* < 0.001) had a significantly lower proportion of survival compared to those of TTTS without sFGR. In addition, donor twins of TTTS with sFGR type I had higher survival than those of TTTS with sFGR types II–III (69% vs. 50%; *p* = 0.185), although not significant. Furthermore, survival of recipient twins was similar irrespective of sFGR type (89% (type I) vs. 90% (types II–III); *p* = 1.000).

In the donor twin, univariate logistic regression analyses showed a significant association between three variables and survival at birth: UA AREDF (OR 0.260, 95% CI 0.094–0.719; *p* = 0.009), sFGR (OR 0.156, 95% CI 0.052–0.464; *p* < 0.001), and GA at surgery (OR 1.290, 95% CI 1.063–1.566; *p* = 0.010). However, in the multivariate logistic regression, being sFGR in any of its forms showed less probability for the survival of the donor twin (Table 3). In the recipient twin, univariate logistic regression analyses found no association between the assessed variables and survival at birth.

## 4. Discussion

This study shows that TTTS with coexisting sFGR is a challenging condition with a worse outcome after laser therapy. According to our results, sFGR is significantly, independently, and negatively associated with donor twin survival in over three-quarters of the cases. We observed a prevalence of sFGR in TTTS pregnancies of 46%, which is similar to that reported in the literature [21]. Although proportions of miscarriage, PPROM and delivery before 32 weeks were higher and GA at delivery was lower in TTTS with sFGR, we did not find statistically significant differences among the groups. This implies an inherent risk of fetoscopy that is not influenced by the presence or absence of sFGR. Given that GA at birth and birthweight play a fundamental role in neurodevelopment, an overall risk of preterm delivery before 32 weeks in almost half of the cases is highly relevant. In addition, TTTS with sFGR showed significantly lower survival of both twins and lower overall survival, but similar survival of at least one twin compared to TTTS without sFGR. This is due to a significantly lower survival of the donor twin in the group of TTTS with sFGR and a similar survival of the recipient twin in both groups. Donepudi et al. [22] reported a significantly higher rate of donor demise within 48 h after laser therapy in the group of TTTS with sFGR in comparison with the group of only TTTS (23% vs. 0%). Moreover, Carmant et al. [23] found significantly lower survival in both twins (49% vs. 69%), especially in the donor twin in TTTS with sFGR compared to TTTS without sFGR. In addition, Gibonne et al. [24] described a significantly lower survival of both twins to discharge from the hospital (55% vs. 73%), and a similar survival of at least one twin (88% vs. 91%) in the group with compared to the group without coexistent sFGR.

Given the differences in placental and vascular characteristics of sFGR sub-types, it is important to recognize the heterogeneity of this population and consider each type separately to ensure proper diagnosis and treatment. While discordance of placental territories is minor in type I and extreme in type III, type II shows the fewest and thinnest vascular anastomosis. This is reflected in the different EDF patterns of the donor UA. In spite of this, we decided to group sFGR types II and III due to the small number of cases and their more pathological Doppler pattern. We observed that even TTTS with sFGR type I had a significantly lower survival for the donor twin as compared to TTTS without sFGR. Among the sFGR types, TTTS with sFGR type I showed a non-significantly higher donor survival rate than TTTS with sFGR types II–III. Since this information is new so far, it cannot be compared with other studies. However, it makes sense given that TTTS with sFGR types II–III have greater placental insufficiency. After laser therapy, chronic blood transfusion between the fetuses is interrupted, so that each fetus is completely dependent on its placental territory. Therefore, the risk of intrauterine donor death would be higher in TTTS with sFGR types II and III.

Findings in our entire TTTS population agree with the fact that overall and double-twin survival increased in the last two decades [25]. Previous series evaluating twin survival according to TTTS stage found that TTTS stage III donors (UA AREDF) showed the lowest double survival rate (45%) [26]. However, the latter study did not take sFGR into account in the analysis. In our population, the proportion of TTTS stage III was significantly higher in TTTS with sFGR. This is not surprising since there is an overlap in the diagnostic criteria of both pathologies. Univariate regression analysis showed no association between TTTS stages III–IV and donor survival but between sFGR and donor survival. Multivariate regression analysis showed that only sFGR and its subtypes were independent predictors of donor survival. This could suggest that sFGR is indeed the main prognostic factor for the survival of these twins. If so, the likelihood of improving donor survival in TTTS with sFGR by LPCV would be greatly compromised, as it is unable to correct the disproportion of placental territories.

From a clinical point of view, our data can contribute to more individualized preoperative counseling. Parents should be informed that after laser therapy for TTTS, coexisting sFGR significantly decreased the donor survival rate, which was lower in types II and III. However, pregnancy complications in terms of miscarriage and preterm delivery before 32 weeks were similar to those of TTTS without sFGR.

The strengths of this study are the use of a uniform protocol and the performance of fetal surgeries by only two surgeons, who did all procedures together, which could have a lesser effect on internal validity. In addition, the experience acquired by surgeons during a one-year hands-on training in a high-volume fetal surgery center before starting the study may reduce the effects of the learning curve. Furthermore, analyzing twin survival according to sFGR types is a more practical and realistic approach. We acknowledge that the retrospective design is a limitation. However, since the data were prospectively recorded, the inherent bias in data collection may be lower. Moreover, a relatively small sample size may affect the statistical power of the study. In addition, as a tertiary referral center, our data can lead to sample selection bias.

In conclusion, MCDA twin pregnancies complicated by TTTS and sFGR undergoing laser therapy presented lower overall twin survival, particularly for the donor twin. Survival of the recipient twin, survival of at least one twin, and pregnancy morbidity were not embedded in the coexistence of sFGR. Maternal-fetal specialists and fetal surgeons should consider these findings in parental counseling. Our results need confirmation by prospective studies.

## Figures and Tables

**Table 1:** Maternal characteristics, ultrasound and surgical data of the study population.

	Study Population (*n* = 101)	TTTS with sFGR (*n* = 46)	TTTS without sFGR (*n* = 55)	*p*
Maternal age (years)	33.2 (29.9–35.8)	33.3 (30.3–35.9)	33.1 (29.0–35.6)	0.675
Caucasian	97 (96)	45 (98)	52 (95)	0.624
BMI (kg/m^2^)	25.5 (22.6–29.0)	24.3 (21.9–28.1)	25.9 (23.7–29.3)	0.117
Nulliparity	51 (51)	25 (54)	26 (47)	0.479
Anterior placenta	50 (50)	20 (44)	30 (55)	0.268
GA at diagnosis	20.6 (18.2–22.7)	19.3 (17.8–21.8)	21.6 (19.3–23.0)	0.019
TTTS stage				<0.001
I	18 (18)	3 (6)	15 (27)	
II	29 (29)	10 (22)	19 (35)	
III	53 (52)	33 (72)	20 (36)	
IV	1 (1)	0 (0)	1 (2)	
Doppler				
Donor twin UA AREDF	23 (23)	20 (44)	3 (6)	<0.001
Donor twin DV ARF	10 (10)	7 (16)	3 (6)	0.178
Recipient twin UA AREDF	6 (6)	2 (4)	4 (7)	0.686
Recipient twin DV ARF	32 (32)	14 (30)	18 (33)	0.805
EFW (g)				
Donor twin	280 (176–432)	214 (148–316)	361 (256–511)	<0.001
Recipient twin	344 (226–526)	307 (210–481)	425 (273–574)	0.021
EFW percentile				
Donor twin	10 (1–36)	1 (0–3)	29 (16–56)	<0.001
Recipient twin	69 (49–87)	68 (52–85)	69 (47–90)	0.879
Intertwin EFW discordance (%)	19 (11–28)	28 (24–36)	11 (6–17)	<0.001
Cervical length < 25 mm	19 (19)	7 (15)	12 (22)	0.398
GA at surgery (weeks)	20.6 (18.3–22.9)	19.5 (17.8–21.9)	21.6 (19.3–23.0)	0.022
Solomon technique	65 (64)	26 (57)	39 (71)	0.133
Duration of surgery (min)	35 (28.5–45.0)	40 (30.5–48.7)	35 (26.5–43.0)	0.013

Data expressed as median (interquartile range p25–p75) or *n* (%). TTTS twin-to-twin transfusion syndrome; sFGR selective fetal growth restriction; BMI body mass index; GA gestational age; AREDF absent and/or reversed end-diastolic flow; ARF absent or reversed flow; EFW estimated fetal weight.

**Table 2:** Pregnancy outcomes and twin survival of the study population.

	Study Population (*n* = 101)	TTTS with sFGR (*n* = 46)	TTTS without sFGR (*n* = 55)	*p*
Miscarriage	4 (4)	3 (7)	1 (2)	0.328
PPROM < 32 weeks	38 (38)	22 (48)	16 (29)	0.053
Preterm delivery < 32 weeks	49 (48)	24 (52)	25 (45)	0.501
GA at delivery (weeks)	32.0 (28.7–34.9)	31.6 (28.5–35.0)	32.9 (29.0–34.9)	0.628
Cesarean section	79 (78)	32 (70)	47 (85)	0.054
Survival at birth				
At least one twin	95 (94)	43 (94)	52 (95)	1.000
Both twins	71 (70)	26 (57)	45 (82)	0.006
Donor twin	78 (77)	28 (61)	50 (91)	<0.001
Recipient twin	88 (87)	41 (89)	47 (86)	0.583
Overall	166 (82)	69 (75)	97 (88)	0.015
BW of live births (g)				
Donor twin	1490 (998–2000)	1300 (932–1922)	1645 (1207–2161)	0.033
Recipient twin	1683 (1208–2238)	1665 (1215–2185)	1620 (1200–2290)	0.854
BW percentile of live births				
Donor twin	32 (13–55)	14 (7–24)	45 (28–63)	<0.001
Recipient twin	53 (31–53)	47 (34–73)	52 (27–77)	0.622
Intertwin BW discordance of living twin siblings (%)	10 (6–20)	20 (6–33)	8 (6–14)	0.002
Neonatal survival				
At least one twin	93 (92)	41 (89)	52 (94)	0.463
Both twins	64 (63)	22 (48)	42 (76)	0.003
Donor twin	72 (71)	24 (52)	48 (87)	<0.001
Recipient twin	85 (84)	39 (85)	46 (84)	0.875
Overall	157 (78)	63 (68)	94 (85)	0.004

Data expressed as median (interquartile range p25–p75) or *n* (%). TTTS twin-to-twin transfusion syndrome; sFGR selective fetal growth restriction; PPROM, preterm premature rupture of membranes; GA gestational age; BW birthweight.

**Table 3:** Multivariate logistic regression of predictors for donor survival.

	OR	95% CI	Adjusted *p*
Donors with and without sFGR (*n* = 101)			
UA AREDF	0.559	0.174–1.800	0.330
sFGR	0.230	0.069–0.766	0.017
GA at surgery	1.221	0.998–1.493	0.052
Donors with sFGR type I and without sFGR (*n* = 81)			
sFGR type I	0.246	0.070–0.864	0.029
GA at surgery	1.105	0.864–1.414	0.426
Donors with sFGR types II–III and without sFGR (*n* = 75)			
sFGR types II–III	0.120	0.033–0.443	0.001
GA at surgery	1.187	0.940–1.497	0.149

OR odds ratio; CI confidence interval; sFGR selective fetal growth restriction; AREDF absent and/or reversed end-diastolic flow; GA gestational age.

## Data Availability

The data that support the findings of this study are available from the corresponding author upon reasonable request.
